# Estrogen regulates muscle bioenergetic signaling

**DOI:** 10.18632/aging.101380

**Published:** 2018-02-04

**Authors:** Eija K. Laakkonen, Rabah Soliymani, Maciej Lalowski

**Affiliations:** 1 Faculty of Sport and Health Sciences, University of Jyväskylä, Jyväskylä, FIN-40014, Finland; 2 Helsinki Institute for Life Science (HiLIFE) and Faculty of Medicine, Biochemistry/Developmental Biology, Meilahti Clinical Proteomics Core Facility, University of Helsinki, Helsinki, FI-00014, Finland

**Keywords:** mitochondrial function, muscle proteome, menopause, estrogenic regulation

Aging *per se* is associated with decrements in skeletal muscle force, power and mass that can be attenuated but not completely prevented by exercise training. Female aging is characterized by menopausal hormonal change in middle-age, which is accompanied by acceleration in muscle decrements, partially attenuated by estrogen supplementation [1]. Despite the presence of well documented data demonstrating that muscle cells express both estrogen and androgen receptors enabling anabolic receptor-dependent signaling, the research focused mostly on male/androgen signaling pathways with less input resulting from female-specific aspects of aging. Amassing evidence implicates estrogens, in particular the biologically most active estradiol (E_2_) that is lost in menopause, to be also involved in the regulation of several aspects of mitochondrial function, including biogenesis, bioenergetics, mitochondrial fusion and fission processes, mtDNA transcription and apoptosis [2].

Despite intensive research efforts following the finding of the role of sex-difference in the pace of muscle aging [3], the molecular mechanisms explaining such difference are not yet fully understood. Omics approaches, covering the molecular picture and including investigations on RNA, proteome and metabolome levels are desired to facilitate a comprehensive view of plethora of various responses to hormonal aging. To complement the previous research on the female muscle proteome [4,5], our recent study focused on proteome level differences evoked by menopause-associated hormonal differences. We utilized *vastus lateralis* muscle samples of premenopausal (30- to 34-year-old) women in comparison with postmenopausal (54- to 62-year-old) monozygotic co-twin women whom the other sister had never been exposed to and the other was a current hormone therapy user (HRT) [6]. The HRT medications used by the study participants involved estrogen only, estrogen and progestogen or tibolone based HRT.

Using explicitly developed sample preparation allowing for efficient solubilization of insoluble muscle proteins combined with label free tandem mass spectrometry (nano-LC-HD-MS^E^) and bioinformatics, we identified in total 1583 and quantified 797 muscle proteins. The quantified proteins were subjected to bioinformatic filtering (fold change above 1.5, P < 0.05, quantified on ≥ 2 unique peptides), to identify differentially expressed proteins (DEPs) amid postmenopausal and premenopausal women. The non-users of HRT vs. premenopausal women comparison yielded 21 DEPs specific to aging coupled with low systemic E_2_ levels. The comparison of HRT users vs. premenopausal women revealed 58 DEPs related to aging with E_2_-supplementation while 93 proteins were shared among both conditions. Furthermore, to identify HRT-use-associated DEPs at genetically controlled, same age background postmenopausal HRT users were compared to their non-using co-twins resulting in the identification of 53 DEPs and 83 additional ones that were specific to the use of tibolone HRT. The two E_2_-containing medication types were combined, while tibolone based HRT was investigated separately due to its known multiple effects including estrogenic, progestogenic and androgenic functions. The intra-pair differences in systemic hormones and in muscle performance characteristics did not differ among co-twin pairs using two different types of E_2_-containing HRT. On the contrary, the intra-pair difference of systemic hormones and specific muscle force (knee-extension force/muscle mass) were significantly different within co-twin pairs using tibolone than in co-twin pairs using E_2_-HRT.

Functional annotation of the DEPs was performed using Ingenuity Pathway Analysis software. In total, 39 significantly affected canonical pathways were found in one or more comparisons. The contributing proteins formed three major functional clusters encompassing mitochondrial functions, cytoplasmic energy metabolism, and cellular signaling, adding further insight to the accumulating evidence that E_2_ participates also in muscle in the regulation of metabolism and mitochondrial activities including bioenergetics and apoptosis [2].

**Figure 1:**
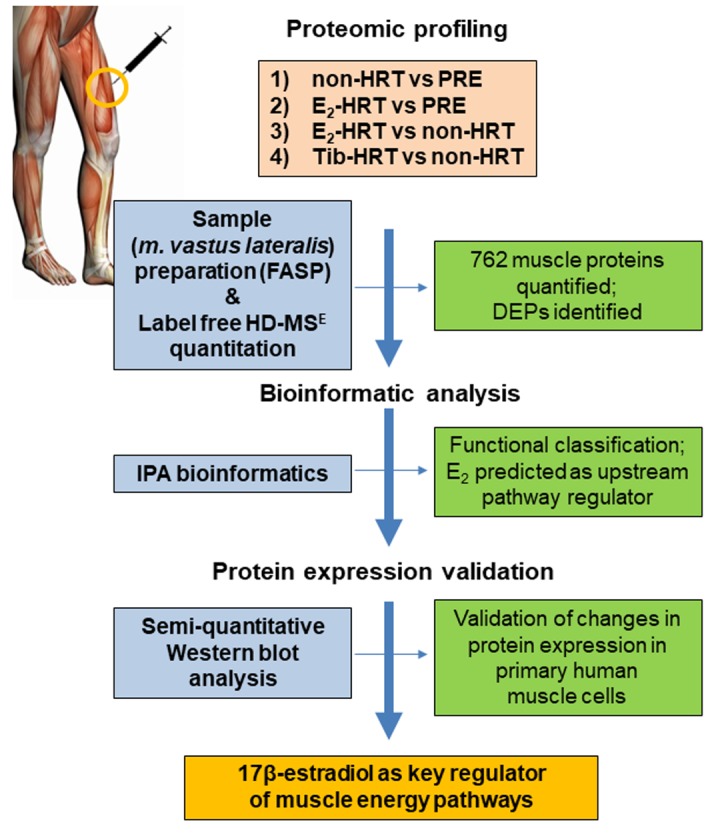
**Outline of proteomics investigations in the female muscle.** Non*-*HRT: postmenopausal non*-*hormone users, PRE: premenopausal women, E_2_*-*based HRT: postmenopausal E_2_*-*HRT users, Tib*-*HRT*-* postmenopausal: Tibolon-based HRT users, FASP: filter assisted sample preparation, IPA: Ingenuity Pathway Analysis, DEPs: differentially expressed proteins.

Based on the observed alterations in muscle protein levels, it appeared that E_2_ constituted a major upstream pathway regulator and pinpointed downstream functions, cell death and glycolytic cascades, to be affected. This complemented the canonical pathway analyses identifying glycolysis/gluconeogenesis as well as oxidative phosphorylation/mitochondrial dysfunction to be influenced by aging and the use of HRT. Taken together, these data collectively implicate E_2_ as a major regulator of muscle cellular function contributing to aging-associated decrement in muscle physiology and performance (Figure 1).

Our results on estrogenic regulation of muscle signaling will stimulate in-depth investigations on mechanisms of hormonal aging and its consequences on muscle aging. Furthermore, our findings bring insights into the regulatory effects of estrogen and the potential of HRT to mitigate the degree of muscular aging. Moreover, the strategy of which type of HRT to prescribe for a woman who is seeking help to control menopausal symptoms is of clinical importance. By providing evidence, for the first time at the human proteome level, that E_2_ is a major regulator of different trajectories of the muscle energy pathways our study is relevant to aging women’s health and may likely be utilized in future interventions aiming at prevention or treatment of menopause-associated metabolic dysregulation, linked to higher prevalence of the cardio-metabolic diseases among postmenopausal women [7].
